# Addressing burn-injury stigma through health education and communication campaign in Pakistan: study protocol

**DOI:** 10.3389/fpubh.2025.1439214

**Published:** 2025-10-24

**Authors:** Özlem Eylem-van Bergeijk, Ali Hussain, Maria Panagioti, Ameer B. Khoso, Zainab F. Zadeh, Muhammad Rehan, Tayyeba Kiran, Alexander Hodkinson, Duolao Wang, Amy Blakemore, Helen Brooks, Tariq Iqbal, Irfan Ullah, Muhammad Mustehsan Bashir, Samia Tasleem, Aamna Sanober, Nabila Soomro, Rakhshi Memon, Nusrat Husain, Nasim Chaudhry

**Affiliations:** ^1^Division of Population Health, Health Services Research and Primary Care, School of Health Sciences, Faculty of Biology, Medicine and Health, University of Manchester, Manchester, United Kingdom; ^2^Manchester Global Foundation, Manchester, United Kingdom; ^3^College of Communication, University of Sharjah, Sharjah, United Arab Emirates; ^4^Division of Population Health, Health Services Research and Primary Care, Institute for Health Policy and Organisation/Alliance Manchester Business School, Manchester, United Kingdom; ^5^Pakistan Institute of Living and Learning, Karachi, Pakistan; ^6^Department of Burn and Reconstructive Surgery, Burn Care Centre, Pakistan Institute of Medical Sciences, Shaheed Zulfiqar Ali Bhutto Medical University, Islamabad, Pakistan; ^7^Department of Clinical Sciences, Liverpool School of Tropical Medicine, Liverpool, United Kingdom; ^8^Mental Health Research Group, Division of Nursing, Social Work and Midwifery, School of Health Sciences, University of Manchester, Manchester, United Kingdom; ^9^Department of Burns and Plastic Surgery Centre, Peshawar, Pakistan; ^10^Department of Plastic, Reconstructive Surgery and Burn Unit, King Edward Medical University Lahore, Lahore, Pakistan; ^11^Burns Center, Dr Ruth K.M. Pfau Civil Hospital Karachi, Dow University of Health Sciences, Karachi, Pakistan; ^12^Department of Plastic Reconstructive Surgery, and Burns, Liaquat University of Medical and Health Sciences, Jamshoro, Pakistan; ^13^Sindh Institute of Physical Medicine and Rehabilitation, Karachi, Pakistan; ^14^Department of Science and Technology Studies, University College London, London, United Kingdom; ^15^Division of Psychology and Mental Health, University of Manchester, Manchester, United Kingdom; ^16^Global Centre for Research on Mental Health Inequalities, Mersey Care NHS Foundation Trust, Liverpool, United Kingdom

**Keywords:** burn survivors, mental health, health education, Photovoice, Pakistan, social media campaigns

## Abstract

**Background:**

Burn injuries remain a neglected public health issue in low-and middle-income countries (LMICs). Up to 90% of burn injuries occur in LMICs, where there is a lack of appropriate burn prevention, acute care and rehabilitation services. When co-created with target populations, health education and communication campaigns could play a pivotal role in promoting risk assessment and improving knowledge, attitudes and practices regarding first aid for burns in low-resource settings. Photovoice is a co-creation approach that engages target communities in capturing and communicating their lived experiences through photography. The present study protocol describes the design of a health education and communication campaign incorporating Photovoice to improve knowledge, attitudes and practices regarding burn injuries in Pakistan.

**Methods and analysis:**

This cohort study will follow 600 participants (*n* = 600; *n* = 500 burn survivors; *n* = 100 caregivers) with three measurement points at 6- to 12-month intervals. The primary outcomes are knowledge, attitudes and practices regarding burn injuries. The secondary outcomes are meaning in life; gratitude, resentment and appreciation; hope; optimism; temporal satisfaction with life; coping strategies; social interaction anxiety; self-stigma and resilience, measured using the Meaning in Life Questionnaire, the Gratitude, Resentment and Appreciation Scale, the Adult Workers Hope Scale, the Patient Optimism Scale, the Temporal Satisfaction with Life Scale, the COPE Inventory, the Social Interaction Anxiety Scale, the Self-Stigma Scale and the Brief Resiliency Scale, respectively. The participants are grouped as follows: the exposure group (i.e., burn survivors [*n* = 200] and caregivers [*n* = 100] exposed to campaign messages), the non-exposure group (i.e., burn survivors [*n* = 250] not exposed to campaign messages) and the Photovoice group (i.e., burn survivors [*n* = 50] who participate in Photovoice and are exposed to the campaign messages). This design evaluates the impact of the health communication campaign and determines whether Photovoice has any additional effects on the primary and secondary outcomes.

**Ethics and dissemination:**

Ethical approval was obtained from the Pakistan National Bioethics (Approval ID: NBC-985) and Manchester University Research Ethics Committees (Approval ID: 2023-18,027-31199). The results of this study will be distributed through public dissemination events, social media channels and peer-reviewed journals.

## 1 Introduction

Globally, burn injuries are one of the top 10 causes of trauma and a leading contributor to premature mortality and years lived in disability (1). The World Health Organization states that approximately 11 million burns and 265,000 burn-related deaths contribute annually to what has been described as a ‘forgotten public health crisis’ (2). Additionally, up to 90% of burn cases occur in low- and middle-income countries (LMICs), where burn prevention, acute care and rehabilitation services are inadequate (2). More strikingly, young girls in LMICs are at higher risk of burns at home, particularly in the kitchen, with fire-related burns being the sixth leading cause of death among females aged 15 and 29 years (2, 3). Furthermore, data obtained from the Global Burden of Disease (GBD) 2019 study indicated that Asia had the most new burn cases in 2019, with a 19% increase relative to cases in 1990 (3,913,524.80 [95% UI 2,946,199.45–4,959,607.51]) (4). Additionally, Asia had the highest death cases in 2019 (57,202.37 [95% UI, 41,804.00–70,564.74]) (4). Even though over half of the global burn-related deaths are estimated to be in South Asia (3), burn injury is an under-researched topic in Pakistan (5). There is a lack of national high-quality epidemiological data representing the remote areas of Pakistan (5). Therefore, the existing epidemiological data is likely underestimate the true health burden in LMICs in general and Pakistan specifically, where injury surveillance systems are rare (6).

Incidents leading to burn injuries can be accidental and/or intentional (5–7). Domestic and work-related incidents are the main contexts in which intentional dowry burn injuries occur in South Asia (5–7). Dowry is property or money given by the bride’s family to the groom or his family at the time of marriage (8). The practice stems from the lack of economic power of young women in the traditional family system and perpetuates oppression and torture (9). Dowry disputes between families often lead to dowry burn injuries among young brides (9). Burn injuries are intentionally inflicted upon the young bride by the groom and/or his family by throwing acid or other corrosive substances on the bride (9). A lack of knowledge of safe work techniques, first aid, illiteracy, poor socioeconomic conditions and the dowry practice are contributing factors (5–9).

Burn injuries affect not only the individual but also their family and community. Burn survivors experience long-term physical, psychological and social consequences (8, 10, 11). Longitudinal and experimental studies indicate that preexisting mental health conditions in both burn survivors and caregivers are closely related to survivors’ rehabilitation and psychological outcomes (12, 13). Many burn patients experience functional limitations shortly after the burn incident, limiting their ability to return to work or daily activities (11, 14). Burn incidents also affect patients’ families. Both patients and family members are at risk of developing post-traumatic stress disorder (PTSD) and experiencing guilt, stigma and discrimination, thus adversely affecting their quality of life (8, 11, 14). In countries where physical appearance is highly valued, burn survivors are often subject to harassment and negative, judgmental words (15). They experience increased distress in response to burn scars and other physical changes, leading to lower negative body image, self-esteem and social isolation (15).

There have been significant advancements in clinical burn care, the successful implementation of preventative initiatives and increased public awareness in high-income countries (16, 17). These advancements have led to a significant decrease in burn injuries and an increase in appropriate, high-quality care (16). Nonetheless, many LMICs still lack effective preventive measures, leading to persistently high rates of burn injuries (18–20). These injuries often occur without access to even the most fundamental health care, which can lead to avoidable fatalities, disabilities and social stigmatisation (18–20). In Pakistan, despite a population of more than 225 million and a high estimated incidence of burn injuries, insurance coverage and rehabilitative services are limited; this includes inadequate initial management of burn wounds and insufficient mental health care, leading to high rates of post-burn complications, such as contractures, PTSD and Common Mental Disorders such as anxiety and depression (21). Additionally, there is a lack of awareness of burn incidents and the challenges that burn survivors and their caregivers endure (21, 22).

Social media has the potential to play a pivotal role in raising public awareness and providing critical information that can aid prevention, improve mental health and reduce stigma (23–25). Recent campaigns in high-income countries show the feasibility of social media (i.e., Facebook, Instagram and TikTok) as an ideal platform for engaging with a wide range of audiences and for delivering public health messages (24). In LMICs, health communication interventions incorporating mass media communication and visual elements demonstrate improvements in knowledge related to burn safety and first aid (3). Additionally, community-based health education sessions consisting of interactive workshops and consultations with the target communities have been shown to be feasible and acceptable strategies for improving knowledge of first aid and prevention in rural areas in Bangladesh (10, 19). Pakistan is an ideal location to implement a health education campaign incorporating social media, mass media communication and community health education sessions. There were approximately 71 million social media users in Pakistan as of January 2022, equivalent to 31% of the total population (25). Furthermore, evidence from a large-scale randomised control trial (RCT) (*N* = 18,554) evaluating the impact of a health education intervention on heat-related outcomes and literacy demonstrated the feasibility and effectiveness of the intervention. The intervention was found to be effective in reducing hospital visits and enhancing heat literacy when compared to the control group receiving usual care in Pakistan (26).

Although education–media interventions show promise in reaching large populations in low-resource contexts and improving knowledge-related outcomes, several limitations restrict their impact. Most are small educational initiatives and lack sufficient long-term follow-ups (3). There is also little focus on involving the target communities during the process of developing and implementing such campaigns (3). To increase the reach and impact of health communication interventions, campaigns must be relevant, appropriate and implemented in collaboration with the target population (27). Hence, co-creation enables researchers to establish partnerships with community members and stakeholders, working together to identify research priorities throughout the process (27). This approach yields data that reflect community experiences, ensuring appropriateness and meaningful impact (27). This is essential to improve knowledge related to burn safety and reduce burn-related stigma globally (23).

One such co-creation approach is Photovoice (28). This method uses photography as a powerful channel to communicate individuals’ lived experiences to a broader audience and destigmatise chronic health and mental health conditions (28–32). Recent meta-analytic evidence of the health care literature showed that Photovoice is especially effective in improving health knowledge among ethnic minority groups in high-resource settings (32). Empirical evidence suggests that when Photovoice was delivered through Instagram, there were both significant immediate and long-term improvements in meaning, appreciation of others and life satisfaction (33). Photovoice has been implemented in high- and low-resource settings to explore the drivers of burn injuries and post-burn experiences of burn survivors (15). Overall, the existing literature highlights the flexibility of Photovoice as a community-based participatory approach applicable to various health conditions in different settings (15). More importantly, Photovoice helps identify the impact of the broader social context, such as stigma, on participants’ experiences; it highlights how social awareness of burn survivors’ needs and burn first aid knowledge is crucial for preventing burn incidents and addressing stigma at a societal level (15). Therefore, it is essential to design a health communication intervention to increase awareness, prevent burn cases and reduce negative social responses in order to improve the mental, emotional and physical quality of life of burn survivors and their relatives in Pakistan. Importantly, burn-mitigating techniques should focus on applying tried-and-tested solutions to promote risk assessment in several settings, such as households, schools and workplaces. These interventions should also improve knowledge, attitudes and practices regarding first aid for burns in LMICs in general and in Pakistan in particular.

The present study is one of the work packages of a larger research programme entitled ‘Burns rehabilitation: A multidisciplinary programme for burns management, treatment and prevention in low-income countries. The project is funded by the National Institute of Research (NIHR) under NIHR Research and Innovation for Global Health Transformation Call 4. The work packages (WPs) of the programme are as follows: understanding the context and intervention co-development (WP1); development and implementation of a national burn registry (WP2); clinical and cost-effectiveness evaluation of the burns care and rehabilitation programme in Pakistan compared to usual care (WP3); development and evaluation of a social media campaign to promote burn prevention and reduce burn-related stigma (WP4); engagement with key informants and policymakers to better understand, prevent and manage burn-related injuries (WP5); and development of sustainable capacity and capability in treatment and rehabilitation for burn injuries and research (WP6). The proposed health education social media campaign, informed by the WP1 findings, will be embedded within WP5 and WP6 to guide policies on burn prevention and management, along with initiatives for capacity and capability building in Pakistan.

The study uses Photovoice to develop and evaluate a collaborative, population-level health education and communication campaign aimed at improving burn prevention and first aid knowledge while reducing burn-related stigma for burn survivors in Pakistan. The campaign will be disseminated through social media platforms to increase its reach and scalability in Pakistan. The present campaign will be the first to evaluate the immediate, medium and long-term effects of the initiative on burn-specific knowledge and on the psychological well-being (meaning, gratitude, life satisfaction, resilience, etc.) of the participants (burn survivors, caregivers, health and emergency care professionals and key informants) in the Photovoice process and subsequent interventions.

The main objectives of this study are as follows:

- Co-create a health education and communication campaign with the target population through Photovoice and health education sessions
- Assess the short- and long-term effects of the campaign on improving knowledge, attitudes and practices regarding burn injuries in Pakistan
- Assess the preliminary effects of Photovoice on improving knowledge, attitudes and practices regarding first aid and on reducing stigma toward burn injuries among burn survivors
- Promote burn prevention and improve risk assessment in workplaces/factories through the health education and communication campaign

## 2 Methods

### 2.1 Research design

**Figure 1:**
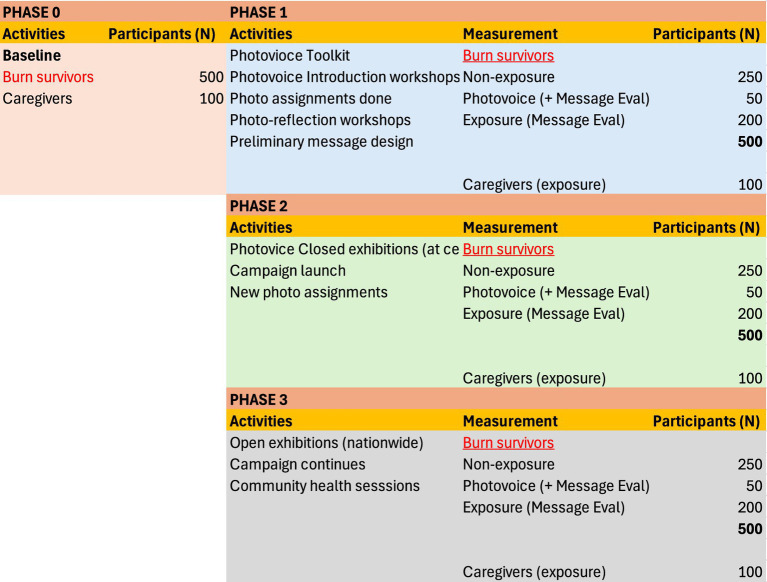
The process of the health communication campaign.

This study is a cohort study with three measurement points conducted at 6- to 12-month intervals (*n* = 600). The impact of the health communication campaign will be evaluated by measuring indicators of knowledge, attitudes and practices related to burn first aid, as well as psychological variables and health information-seeking variables, among the following groups: the exposure group (i.e., burn survivors [*n* = 200] and caregivers [*n* = 100] exposed to campaign messages), the non-exposure group (i.e., burn survivors [*n* = 250] not exposed to campaign messages) and the Photovoice group (i.e., burn survivors [*n* = 50] who participate in Photovoice and are exposed to the campaign messages). Figure 1 presents the campaign process and the planned activities in different phases.

The study is scheduled to take place from March 2024 to October 2026 at 12 participating centres in seven research sites (Lahore, Karachi, Islamabad, Multan, Quetta, Hyderabad and Peshawar) in Pakistan.

### 2.2 Phase 0: baseline assessment

The research team will recruit participants (*n* = 600) from seven project sites across Pakistan for a baseline assessment measuring existing indicators of knowledge, attitudes and practices on burn prevention, psychological variables, health information-seeking variables and demographics (see Table 1 for the full list of variables).

**Table 1:** Outcome measures collected at the data collection points throughout the study.

Outcome measures	Baseline (t0)	Time 1 (t1)	Time 2 (t2)
KAP ^1,2^	X	X	X
SSS-S^1^	X	X	X
GRAT^1^	X	X	X
MLQ^1^	X	X	X
WHS^1^	X	X	X
CPOS^1^	X	X	X
TSWLS^1^	X	X	X
COPE^1^	X	X	X
SLAS^1^	X	X	X
BRS^1^	X	X	X
Photo Reflection^1,2,3,4*^		X	X
Campaign effectiveness^1,2,3,4^			X

Survivors^1^; family members ^2^; health care professionals^3^; key informants^4^; the photo reflection workshops are for burn survivors only except for the workshops conducted at the end of the public exhibitions^*^; t0, baseline; t1, end of photo reflection workshop; t2, end of closed exhibitions; t3, end of closed exhibition; t4, end of the final public project exhibition; KAP, Knowledge Attitude and Practice Scale; MLQ, Meaning in Life Questionnaire; GRAT, Gratitude and Resentment Scale; WHS, Adult Workers Hope Scale; CPOS, Patient Optimism Scale; TSWLS, Temporal Satisfaction with Life Scale; COPE, COPE Inventory; SIAS, Social Interaction Anxiety Scale; SSS, Self-Stigma Scale; BRS, Brief Resiliency Scale.

Ethical approval was obtained from the Pakistan National Bioethics (Approval ID: NBC-985) and Manchester University Research Ethics Committees (Approval ID: 2023–18,027-31199).

#### 2.2.1 Sampling

A combination of snowball and purposive sampling methods will be used to identify and recruit patients (burn survivors), caregivers and key stakeholders (i.e., health and emergency care professionals and key informants, including community leaders and policymakers), based on the eligibility criteria described below.

#### 2.2.2 Participants

A wide range of participants will be recruited for the study. As the media interventions vary in scope and objectives, not all participants will receive the same questionnaires and Photovoice intervention. Below, we describe the scales to be administered to each participant.

#### 2.2.3 Procedure

The research team will recruit burn survivors and caregivers from 12 burn centres based in seven project sites across Pakistan to measure existing indicators of knowledge, attitudes and practices on burn prevention, as well as psychological variables, health information-seeking variables and demographics.

**Figure 2:**
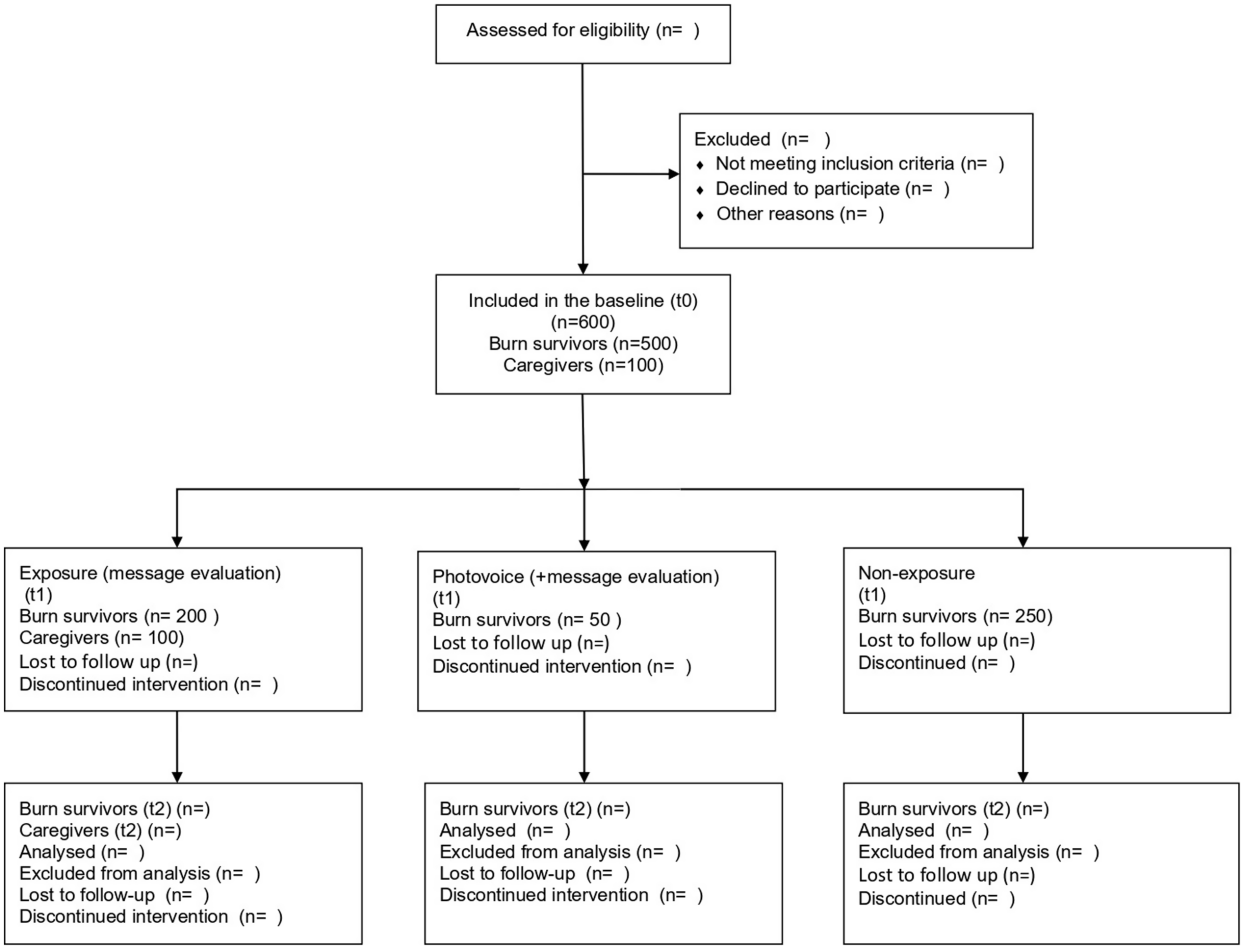
CONSORT diagram.

Burn survivors and their family members will be invited to fill out online questionnaires (baseline assessment). The research assistants will distribute posters and leaflets at participating centres and waiting rooms, introducing the study and providing the research team’s contact information for further enquiries. The research assistants will also attend the patient and/or family consultations to introduce the study and obtain consent for a follow-up call with the research team. During the follow-up call (within the first 24 or 48 h after the initial contact), the research assistants will ensure that interested burn survivors and their family members understand what their participation entails, and will confirm their willingness to provide informed consent. After informed consent is obtained, burn survivors and their family members will be asked to complete the same questionnaires at 6- to 12-month intervals up to a total of two times throughout the study (see Figure 2 for the CONSORT diagram). If they are literate, as determined during the informed consent process, they will receive a text message with a Qualtrics questionnaire link. If they are illiterate, a research assistant from the research team will call them for the completion of the questionnaires on the phone. Consent for collecting personal information will be obtained during the informed consent process.

The rest of the participant groups, including key stakeholders (i.e., health and emergency care professionals) and key informants (i.e., community leaders and policymakers), will be recruited purposively for the development and implementation of the social media and health communication campaign (see Figure 1 for the project activities). They will provide input on the content of the campaign messages, dissemination and campaign reach.

#### 2.2.4 Eligibility criteria

##### 2.2.4.1 Burn survivors

The following are the inclusion criteria for burn survivors:

- Adults aged 18 years and above
- Recent (i.e., within the past 3 months) and/or past experience of a burn injury
- Resides in one of the seven research sites
- Able to provide consent and participate in the mixed methods study, which requires their engagement in project activities, including surveys, Photovoice reflection workshops and closed/open exhibitions depending on the group to which they are assigned
- For those involved in the Photovoice group, an additional criterion includes willingness to engage with participatory creative methods, such as photography and storytelling, as well as availability to attend a minimum number of Photovoice workshops.

The following are the exclusion criteria:

- Burn survivors with intentional and/or self-inflicted injuries will be signposted to the available services for further help and will not be included in the study.

##### 2.2.4.2 Caregivers

- Adult family member (age 18 years and above) of a burn survivor or of those who passed away as a result of a burn injury
- Able to provide consent and participate in the study

##### 2.2.4.3 Health and emergency care professionals

- Possess experience in managing burn cases
- Currently working in a burn care centre and/or emergency care

##### 2.2.4.4 Key informants

- This will include community leaders and policymakers with exposure to and an understanding of burn prevention and burn care in Pakistan

#### 2.2.5 Measures

Qualtrics software will be used for the online questionnaires. These questionnaires will be available in English, Urdu and/or other local languages of the participants (56, 57). A self-developed demographic questionnaire will be used to obtain information on the participants’ gender, age, level of education, marital status, native language, place of residence (rural and/or urban), employment status, self-reported general health and other variables. A unique tracking code will be developed to monitor each participant between measurements.

##### 2.2.5.1 Knowledge Attitudes And Practice (KAP) Scale

The KAP includes a range of structured interviewer-administered questions to obtain a better understanding of knowledge, attitudes and practices regarding burn injuries (22, 34). The knowledge- and attitude-based questions include 10 items, scored on a four-point Likert scale ranging from strongly disagree to strongly agree. Higher scores represent better knowledge of and favourable attitudes toward burn injuries and first aid. The practice of burn first aid includes 12 questions with various response formats, such response categories such as ‘true/false’ and ‘yes/no’. A sample item for the knowledge-based questionnaire is, “Burns lead to permanent injuries?’ A sample item for the attitude-based questionnaire is, ‘Do you think that applying dough, oil, mud, toothpaste, etc. on the wound delays the healing process?’ A sample item for the practice of burn first aid is, ‘In case of a burn injury, have you ever applied cold water?’ The internal consistency, measured using Cronbach’s alpha, was 0.887 for the knowledge-based questionnaire, 0.716 for the attitude-based questions and 0.758 for the practical questions (22).

##### 2.2.5.2 Self-Stigma Scale (SSS)

The SSS (35) has both long (39 items) and short (9 items) versions, comprising three self-stigma subscales (cognitive, affective and behavioural), and is used to assess internalised stigma. In this scale, respondents rate their levels of agreement with each item (e.g., ‘My identity as a ______is a burden to me’ [cognitive], ‘I fear that others would know that I am a_____’ [affective], and ‘I estrange myself from others because I am a____’ [behavioural]) on a four-point Likert-type scale (1 = strongly agree to 4 = strongly disagree). The blank spaces on the scale were filled with ‘mental health consumer’ when used in a sample of mental health consumers and with ‘recent migrant’ when used in a sample of migrants (35). In this study, the blank spaces will be filled with the term ‘burn survivor’ to measure internalised stigma associated with this identity. Higher scores indicate greater self-stigma. Internal consistency estimates ranged from 0.87 to 0.91 for the total scale and from 0.80 (behavioural) to 0.84 (affective) for the subscales (29). Mean scores between 2.40 (SD = 0.66) and 2.41 (SD = 0.60) reflect moderate to high internalised stigma (35). More recently, the reliability of a shorter version of the scale (5 items) has been established, with Cronbach’s alphas of 0.93 and 0.91; it has demonstrated adequate convergent and criterion-related validity in an English-speaking sample in the US (36).

##### 2.2.5.3 Meaning in Life Questionnaire (MLQ)

The Meaning in Life Questionnaire (MLQ) (37) assesses self-reported experiences of the presence of meaning and the search for it. The questionnaire consists of 10 items rated on a seven-point Likert-type scale ranging from 1 (absolutely untrue) to 7 (absolutely true). Some examples of items are ‘I have a good sense of what makes my life meaningful’ (presence of meaning) and “I am always searching for something that makes my life feel significant’ (search for meaning). The instrument has been shown to be reliable in various contexts, with Cronbach’s alphas ranging from 0.83 to 0.91 for both subscales (37, 38).

##### 2.2.5.4 Gratitude, Resentment And Appreciation (GRAT) Scale

The GRAT Scale (39) will be used to measure the participants’ overall level of gratitude. This instrument measures participants’ lack of sense of deprivation (LOSD), simple appreciation (SA), and appreciation of others (AO) on a nine-point Likert-type scale ranging from 1 (strongly disagree) to 9 (strongly agree). LOSD is measured by items such as ‘Life has been good to me’, SA by items such as ‘Oftentimes, I have been overwhelmed at the beauty of nature’ and AO by items such as ‘I feel deeply appreciative for the things others have done for me in my life’. Across samples, this instrument has been shown to be reliable, with Cronbach’s alphas ranging from 0.88 to 0.94 on various subscales (39).

##### 2.2.5.5 Adult Workers Hope (WHS) Scale

This scale focuses on work-related goals, pathways and agency (40). The WHS consists of 24 statements to which respondents indicate their degree of agreement. The item response format is a seven-point Likert-type scale ranging from 1 (strongly disagree) to 7 (strongly agree) and with the total scores ranging from 24 to 168 (40). Cronbach’s alpha coefficient was found to be 0.90 (40).

##### 2.2.5.6 Patient Optimism Scale (POS)

The POS was developed based on a cancer-related patient optimism scale and adapted for burn survivors (41). The authors defined optimism as the ‘patient’s belief that he or she had made appropriate choices regarding his or her treatment and the patient’s hopefulness about treatment outcomes’. The scale is part of a measure that assesses patient fortitude, trust in nurses (health care providers) and authentic self-representation. Participants are asked to answer each question using a six-point scale ranging from 1 (never) to 6 (always). Scores are then transformed into a 0–100 range, with higher numbers reflecting higher levels of optimism (41). The Cronbach’s alpha coefficient for the scale was 0.75, and the item–scale correlations ranged from 0.44 to 0.65 (41).

##### 2.2.5.7 Temporal Satisfaction with Life Scale (TSWLS)

The TSWLS measures three temporal dimensions of life satisfaction: past, present and future (42). It consists of 15 items assessing individuals’ global life satisfaction, separated based on three temporal dimensions (i.e., past, present and future satisfaction). Respondents rate their agreement with each item on a seven-point Likert-type scale ranging from 15 (strongly disagree) to 75 (strongly agree). All 15 items are positively keyed. Four scores can be obtained for the analyses. First, summing all 15 items leads to a total score ranging 15 to 105. Second, summing the five items for each temporal dimension (past, present and future satisfaction) provides three subscale scores ranging from 5 to 35 (42).

##### 2.2.5.8 COPE Inventory

The COPE Inventory is a measure of dispositional tendencies to adopt particular coping styles (43). The scale identifies the personality characteristics that might predispose individuals toward a particular way of coping with stress. The COPE Inventory is a 60-item measure comprising 15 subscales with four items each. The subscales are planning, active coping, suppression of competing activities, restraint, use of instrumental support, use of emotional support, positive reinterpretation and growth, acceptance, religious coping, focusing on and venting emotions, denial, behavioural disengagement, mental disengagement, substance use and humour. Individuals are asked to indicate the extent to which they use these coping strategies when encountering difficult or stressful events in their lives. The dispositional COPE Inventory coefficients ranging from 0.45 (mental disengagement) to 0.92 (turning to religion) in a large sample of 978 undergraduates (43).

##### 2.2.5.9 Social Interaction Anxiety Scale (SIAS)

Social interaction anxiety refers to distress experienced when meeting and talking to people, specifically ‘fears of being inarticulate, boring, sounding stupid, not knowing what to say or how to respond within social interactions, and being ignored’ (44). We have included this measure of social anxiety because levels of social anxiety reflect the degree to which people are motivated to make impressions on others but doubt that they will make the impressions they desire (44). Respondents indicate the degree to which each statement is characteristic or true of them on a five-point Likert-type scale ranging from 0 (not at all) to 4 (extremely). The Cronbach’s alpha coefficients exceeded 0.88 for all five initial samples (44, 45). Inter-item reliability exceeded 0.85 (46).

##### 2.2.5.10 Brief Resiliency Scale (BRS)

The BRS is a six-item self-report scale (47). Sample items include the following: ‘I tend to bounce back quickly after hard times’; ‘I have a hard time making it through stressful events’; ‘It does not take me long to recover from a stressful event’; and ‘It is hard for me to snap back when something bad happens’. The response options for all items are ordered on a five-point Likert-type scale. The scale demonstrated appropriate internal consistency, with Cronbach’s alphas ranging from 0.80–0.91 across four samples in the study (47).

The sample size calculation for the study is based on a comparison of the baseline and follow-up measures of the primary outcome, knowledge of burn-related prevention, in this cohort study. Using a previous study as a reference (22), we determined that a sample size of 478 is required to achieve a 70% improvement in knowledge of burn-related prevention, assuming a 63% rate of poor knowledge at baseline, a 5% significance level and 91.5% power at the 24-month follow-up (last follow-up). A total of 600 participants will be required, with a 20% dropout rate taken into account.

### 2.3 Phase I: development and implementation of the campaign

#### 2.3.1 Photovoice

##### 2.3.1.1 Sampling

To investigate the preliminary effects of Photovoice on the primary and secondary outcomes of interest, we will categorise participants into three groups, as described previously (see Figure 1). As this is only a pilot study and the objective is mostly to co-adapt the Photovoice programme and evaluate its potential in building capacity for a larger evaluation, no sample size calculation has been deemed appropriate (48, 49). This study will also help us determine whether we can maintain an acceptable attrition rate of Photovoice for a future RCT (50). An effective sample size for this study was determined based on the sample size of previous exploratory studies using Photovoice (31).

Consenting baseline participants will be invited to attend the introductory Photovoice workshop via email and/or a telephone call. The invitation letters will be sent via email and/or postal mail. One-to-one meetings will be organised with those who do not want to be in a group setting.

#### 2.3.2 Procedure

##### 2.3.2.1 Introductory workshops

We will host introductory workshops at the burn and community centres and governmental institutions across Pakistan, where the interviews and focus groups will be held as part of other WP activities. During these workshops, the project will be explained verbally and through the distribution of information leaflets, while any queries will be addressed by the research staff. We will then seek oral and written informed consent and train the participants to use their phone cameras for photography on the same day. Before being disseminated, the training content and the instructions will be pilot tested on a small group of key stakeholders already involved in another WP of the study. Participants without phone cameras will be given disposable cameras and asked to take photos (post-workshop) depicting their realities of being burn survivors and/or having relatives who are burn survivors. Participants will be assured that there are no ‘right’ or ‘wrong’ ways to take a photo and that no professional photographic training will be provided so that the photos reflect the participants’ own views. Once the participants provide their informed consent, Photovoice facilitators (as part of the research team) will call them on a fortnightly basis to check whether there are any safety-related, personal and/or logistical difficulties with taking pictures and whether the participants need additional support. There will be 10 Photovoice facilitators in total, and each facilitator will manage communication and monitor the Photovoice process and safety of five participants from participating centres.

##### 2.3.2.2 Photo reflection workshops

Participants will then be invited to the photo reflection workshops. They will be asked to bring three to five of their photos for individual reflection, which will be guided by the following photo-trigger questions: Describe what you see in this Photo: How does this photo make you feel? Why did you take a photo Of this? What does this photo Tell us about your experience with burns? How can this photo provide Opportunities for us and others to improve our lives. The participants will work individually with the facilitators to produce photo captions. At the end of the workshop, the participants will be asked to fill out a questionnaire package based on the baseline measures again and will be invited to the closed local exhibitions.

##### 2.3.2.3 Closed local exhibitions

Private exhibitions of the photos will be conducted at the participating centres. These exhibitions will be closed to the public for the following reasons: (1) to protect the participants’ identity, (2) to help build their confidence in displaying their photos, (3) to provide an opportunity to revise their photo captions and (4) to sign consent forms to have photos of themselves blurred/unblurred in preparation for public dissemination and exhibitions. The participants will be given 2 weeks to decide, if needed, and the research assistants will follow up with them. The participants will be asked to fill out the same questionnaire package based on the baseline measures at the end of the closed exhibitions.

##### 2.3.2.4 Training and supervision of Photovoice facilitators

The photovoice facilitators will be trained by the corresponding author in collaboration with the Photovoice Worldwide team, which has expertise in developing customised Photovoice training content for research groups and implementing Photovoice projects in both high- and low-resource settings. The training will be organised into two modules: (1) introduction to Photovoice and facilitation and (2) advanced Photovoice training. The first module will cover background information and will introduce basic facilitation skills in the field. It will also identify the need for advanced training of the facilitators. The second module will be more interactive and will mainly focus on role-plays to familiarise facilitators with the possible ethical (i.e., power sharing) and/or cultural challenges encountered (i.e., use of culturally appropriate language while facilitating Photovoice workshops) and how to solve them in the field. Following these training modules, a working group including health care professionals, key stakeholders (i.e., key informants and burn survivors) and WP1, WP3 and WP5 leads will be formed, and a co-designed Photovoice toolkit will be developed. This toolkit will be used as a guide for the facilitators to train the rest of the facilitators. The toolkit will include background information on Photovoice, facilitation principles, dealing with ethical and cultural challenges, supervision and social media guidelines, supplementary materials (e.g., information sheet and consent forms) and distress and debriefing policies for the facilitators. Follow-up training for the facilitators will also be organised depending on the need. Supervision of the facilitators will be managed by the clinical psychologist of the team on a weekly basis. In-person and online group or one-to-one supervision meetings will be organised to discuss the facilitation process and communication between facilitators and burn survivors during the Photovoice process.

### 2.4 Phase III: public dissemination of campaign messages through social media channels and community health education sessions

The priority areas for phase 3 will be determined through the findings from the photo reflection workshops and the interviews social media and health communication campaign content.

In phase 3, the participants (burn survivors) will be given instructions to continue taking photos using their mobile phones after the Photovoice workshops. They will be asked to take pictures of meaningful moments in their daily lives and reflect upon what the photos mean/represent. These instructions will also be pilot tested on a small group of individuals before being disseminated. The participants’ photos from the closed local exhibitions (based on consent) and the new photos that they capture through their mobile phones will be posted on the project website and on social media. There will be a moderation procedure in place. The research assistants will moderate online interactions by monitoring comments and responding to posts. Photos and/or stories with dedicated hashtags will be released at various high-activity times of day. Posters and/or leaflets about the social media accounts will be displayed across public spaces, such as schools and workplaces, to generate more interest in the campaign.

Health messaging will be promoted through organic and paid posts on main social media sites to increase engagement with the audience. A central website will be maintained on WordPress to host the main messages on burn prevention and first aid and will be used as a landing page for the audience to acquire more information. The website will also serve as a central repository for social media content and links for future use. The overall objective is to increase audience engagement with the campaign through likes, comments and shares.

We will run a comprehensive marketing campaign to promote the messages on Facebook, Instagram and Twitter. The promotional content will include video animations, audio/visual stories and infographics. The campaign will promote the content of the campaign and drive traffic to the project website. Broadly, we estimate to reach ~5 million people in Pakistan.

In addition to the online campaign, we will conduct health education sessions for the most vulnerable target audiences at schools, community centres, workplaces, factories and burn centres. These health education sessions will target key informants and stakeholders, aside from burn survivors and their family members. A comprehensive social media messaging campaign will support this in-person communication by providing a centralised location to access more information for burn prevention and first-aid measures. We will also liaise with journalists, TV presenters, radio programmers and bloggers to publicise the campaign. We will ensure that captions to complement photographs, stories and hashtags are culturally sensitive and translated into the study languages.

## 3 End of project exhibitions (nationwide)

Finally, we will organise a series of end-of-project exhibitions across the designated research sites in Pakistan to which a broad range of stakeholders, community workers, carers, health care professionals, policymakers and journalists will be invited. With their consent, these end-of-project exhibitions will provide a platform for the participants and others exposed to Photovoice content to talk about their experiences. We will also liaise with journalists, TV presenters, radio programmers and bloggers to disseminate the exhibitions on a national scale. Participants who took part in the Photovoice process will be asked to fill out the same questionnaire package based on the baseline measures at the end of the public exhibitions.

## 4 Data analysis plan

To compare changes in self-reported outcomes within groups across two time points (see Table 1) in the sample (*N* = 600), we will implement linear mixed-effects models implemented in R (R version 3.6.1) (51) using the lme4 package and in SAS 9.4. This method considers correlations within participants, allowing them to be modelled appropriately. It also estimates within-subject variability, in addition to estimating model parameters. The method can handle and model missing outcome data under the assumption that the data are missing at random. The mixed model uses a longitudinal data structure that includes both fixed and random effects, and baseline measurement is usually included as a covariate. Time will be included in the model as an interacting categorical variable to observe any time-varying trends. Cohen’s *d* for the effect of Photovoice will be calculated as the difference between baseline-corrected estimated means divided by the pooled standard deviation. A two-sided *p* < 0.05 indicates statistical significance. In addition, demographic variables, such as age, education, gender, marital status, geographic location (i.e., rural vs. urban residence) and household situation (i.e., living with a single and/or an extended family) will be included in the linear mixed models to test for potential moderating and mediating effects on behavioural intention related to burn prevention and first-aid practices. A stepwise selection of the variables will be conducted to determine which variables to keep in the model. As we add the variables, we will immediately eliminate those that do not reduce the Akaike information criterion (AIC) by using the *stepAIC* R function from the library *MASS*. If two or more independent variables in a model are found to be strongly correlated, a variance inflation estimate of less than 5 will be used to determine whether collinearity is present. If the relationship is found to be non-linear, attempts will be made to model the outcome data via the use of splines or polynomials in the *nlme* package in R. The normality of residuals will be assessed graphically using histograms and Q-Q plots. Detailed statistical analyses following Good Clinical Practice: Consolidated Guidelines ICH E9 (52) and Medical Research Council guidelines for developing complex interventions (53) will be described in the statistical analysis plan, which will be finalised before the data lock stage.

In line with the flexibility of the Photovoice approach as a participatory action research method, we decided to follow reflexive thematic analysis based on Braun and Clarke’s six-phase approach (54), with NVivo software supporting data management and coding. All reflection sessions and exhibitions will be audio-recorded, transcribed verbatim and pseudonymised. Two researchers will independently code a subset of transcripts to enhance rigour and then collaboratively develop an analytical framework. Both inductive and deductive coding will identify emergent themes. Thematic analysis will be conducted on captions, participant narratives and social media comments. Special attention will be given to potential group differences (e.g., male vs. female participants, urban vs. rural residence, severity of burn injuries, and communication or health literacy differences). Data analysis will occur in parallel with data collection. Thick descriptions of the context of each workshop and the interactions with each participant will be documented in field notes. The team will agree when data saturation has been reached.

Social media metrics and textual analysis of photo captions and viewer comments will be employed to determine audience reach and engagement. These metrics will also provide insights into perceptions, knowledge and attitudes related to burn survivors and their experiences. The analytics dashboards provided by each social platform will be used to gather key metrics. The main metrics include likes, comments, shares and emojis. Specifically, the comments will be used for thematic analysis to extract more narratives, stories and lived experiences. Overall, a triangulation approach will be used to integrate the results from the different data sources, provide a comprehensive overview of the findings and enhance confidence in the conclusions (55).

## 5 Ethical considerations

The study may involve individuals at risk of harming themselves and/or others. Furthermore, it is possible that on some occasions, social media posts draw negative publicity, and some participants might feel distressed and/or upset as a result. To mitigate these risks, we will take the following precautions:

The project will be explained to the participants, and their written and oral informed consent will be obtained. The research staff are fluent in English, Urdu and other local languages of the study sites. They will ensure that the participants are aware of the requirements for taking part in the study and the social media campaign. The participants may withdraw from the study at any time, and any social media posts and/or data they provided will be excluded from the study.

There will be a dedicated Photovoice facilitator managing the communication, Photovoice process and safety of the participants throughout the Photovoice process. Facilitators will call participants on a fortnightly basis to check whether there are any safety-related, personal or logistical difficulties with taking pictures and whether the participants need additional support. In case a participant discloses unpleasant experiences while taking pictures and/or if they mention remembering unpleasant memories about their past accident(s) resulting in burn injuries, the Photovoice facilitator will discuss these with their clinical supervisors; the clinical supervisor from the team will then call the participant to follow up on the issue. The participant will be signposted for further psychological help if needed.

In case a participant discloses thoughts of harming oneself and/or others during the Photovoice workshops and/or on social media platforms, a distress protocol will be used, and it will be individualised for each participant. At the introductory workshops, the participants will be asked to provide the names and contact details of three relatives/friends. If a participant discloses thoughts of harming oneself and/or others at any point, the psychologist from the research team will perform a risk assessment (see exploring risk questions), either in person and/or through a phone call. If deemed necessary, the psychologist will contact the relatives of the participant and take them to a nearby psychiatric unit in a public hospital. As a last resort, hospital emergency services will be contacted if the participants and/or their relatives cannot be reached. In case of an adverse event (i.e., suicide attempt or self-harm), an Adverse Event Form will be completed and sent to the principal investigator within 24 h of reporting the event. Furthermore, if the individual discloses thoughts of self-harm and/or harming others and is a social media follower unknown personally to the research staff, the latter will send a private message with emergency contact numbers and support resources.

Participants may share their photos with the Photovoice facilitators before the photo reflection workshops or prior to the closed and/or open exhibitions. This would be necessary to avoid inconveniences and delays due to the postal system in Pakistan. To protect the participants’ privacy and confidentiality, the authors will provide the participants and the research assistants with mobile phones and will communicate exclusively through these phones (not through their personal mobile phones). The participants’ pictures will be stored on the research assistants’ mobile phones and will be permanently deleted as soon as these are transferred to the data management system at the Pakistan Institute of Living and Learning. The data management system is protected with a username and password, and it is only accessible to the research staff in Pakistan.

A moderation process will be implemented to ensure the safety of the participants throughout the social media campaign. The research staff will manage the social media accounts of the project and will actively monitor comments and respond to posts. Offensive or harmful comments will be deleted, and any users engaging in harassment, will be blocked. If a negative exchange between the participants and followers of the social media accounts occurs about particular photos and/or stories, the psychologist of the research team will contact the participant via telephone and/or email and will assess their psychological distress. If deemed necessary, the participant will be signposted to the available psychological support services in their area.
